# Clinical safety and possible efficacy of tirofiban in combination with intravenous thrombolysis by recombinant tissue plasminogen activator for early treatment of capsular warning syndrome (CWS)

**DOI:** 10.3389/fnins.2022.1026127

**Published:** 2022-11-16

**Authors:** Yunzhu Liu, Shiyong Li, Dongdong Hao, Zhongping Zhang, Yongxin Yi, Jiayang Fang, Weizhi Lin, Min Zhang

**Affiliations:** ^1^Department of Encephalopathy, The Third People’s Hospital Affiliated to Fujian University of Traditional Chinese Medicine, Fuzhou, China; ^2^Department of Encephalopathy, Xiamen Hospital of Traditional Chinese Medicine, Xiamen, China; ^3^Department of Outpatient, Lanzhou 7th Rest Center for Retired Cadre, Lanzhou, China

**Keywords:** capsular warning syndrome, tirofiban, intravenous thrombolysis, acute ischemic stroke, perforator occlusion

## Abstract

The purpose of this study was to assess the efficacy and safety of the combination of tirofiban with intravenous thrombolysis (IVT) in treating patients with capsular warning syndrome (CWS) who failed to respond to the treatment of intravenous thrombolysis alone. Tirofiban was approved for the treatment of CWS patients with fluctuating symptoms or no substantial improvement after intravenous thrombolysis within 24 h in our hospital from October 2019 to June 2021. Patients were evaluated with the National Institutes of Health Stroke Scale (NIHSS) at admission, at 72 h post-thrombolysis, at 1-week, and at 3-months with the modified Rankin Scales (MRS) score. A total of 12 patients received tirofiban and eight patients received control treatment with a history of CWS in our cohort. Among the patients, 13 patients smoked more than one pack of cigarettes a day, 17 had hypertension, 17 had hypercholesterolemia, 7 had diabetes, 1 had the history of cerebral infarction, 2 had atrial fibrillation, 7 had mild big vascular stenosis, 13 had lesions of the perforating branch by imaging, and 19 had acute capsular infarction. In both the tirofiban and control groups, NIHSS scores were significantly reduced after intravenous thrombolysis or 1-week after onset compared with before intravenous thrombolysis (*P* < 0.001). Before and after intravenous thrombolysis, there were no differences between the tirofiban group and control group (*P* = 0.970, *P* = 0.384, respectively). The tirofiban group, however, showed remarkably lower scores in both 1-week NIHSS and 3-month MRS than the control (*P* = 0.012, *P* = 0.003, respectively). Our study revealed that tirofiban did not increase the risk of hemorrhage and had favorable clinical efficacy as a remedial treatment for CWS patients with poor prognosis for intravenous thrombolysis, therefore indicating great potential for broader use.

## Introduction

In Donnan et al. (1993) defined capsular warning syndrome (CWS) as being one of many subtypes of transient ischemic attacks (TIAs). Clinically, it manifests through recurring episodes of stiffness and/or dyskinesia, including sensory anomalies and pure motor hemiparesis. Infarct loci often occur within the capsule rather than in the cortex (Donnan et al., 1993; Staaf et al., 2004), posing a high risk of permanent deficits with poor prognoses. CWS pathogenesis has been more fully understood in recent years thanks to widespread clinical application of imaging technology (Gao et al., 2022). It is generally recognized that this subtype of branch atheromatous disease (BAD) results from an unstable cerebral perfusion pressure leading to hypoperfusion in the branch caused by the presence of atherosclerotic plaque and incomplete infarction of the branch. Thus, it is common to experience TIAs, which may eventually develop into acute ischemic strokes (AIS).

Tirofiban is a glycoprotein inhibitor (GPI) that selectively targets and antagonizes the receptors for platelet GPIIb/IIIa receptor, which is involved in the common downstream signaling pathway that mediates thrombosis and platelet aggregation (Junghans and Siebler, 2003; Yang et al., 2019). The antagonist of GPIIb/IIIa occupies this receptor and prevents its binding to adhesive proteins, thereby inhibiting platelet aggregation (Junghans and Siebler, 2003; Yang et al., 2019). In order to improve the clinical efficacy of CWS, a prospective, open-label, historical controlled, cohort study was conducted to identify that the replacement of tirofiban by intravenous thrombolysis can effectively improve the clinical prognosis for CWS caused by atherosclerotic occlusion of perforating arteries. Furthermore, we summarized preliminary results from subsequent and continuous treatment with tirofiban in poor responders to 24-h intravenous thrombolysis with recombinant tissue plasminogen activator (rt-PA). A comparison of the safety and efficacy of tirofiban with intravenous thrombolysis of rt-PA alone was conducted between the tirofiban and the control groups. Our study highlights the significant clinical effect of tirofiban as a remedial treatment for CWS patients with poor prognosis for intravenous thrombolysis.

## Materials and methods

### Design and objects selection for the present study

This is a prospective, open-label, cohort study that is based on historical controls. Patients in the experimental group were diagnosed with CWS and hospitalized in our facility from October 2019 to June 2021. The patients were given continuous tirofiban treatment immediately when they had no obvious relief of symptoms (reduction of NIHSS score < 4) or symptoms fluctuated after temporary relief (NIHSS score increase > 4) within 24 h of intravenous thrombolysis with rt-PA. The control group consisted of the patients with CWS who were admitted to our hospital from January 2018 to March 2019 and received routine intravenous thrombolysis with rt-PA alone. The National Institutes of Health Stroke Scale (NIHSS) scores before and after intravenous thrombolysis, after symptom fluctuation, 72 h after tirofiban and 1-week after onset and modified Rankin Scales (MRS) scores for 3 months were recorded (Camps-Renom et al., 2015). The adverse events, such as intracranial hemorrhage, systemic hemorrhage, and decreased platelet count during their hospitalization were also monitored and recorded from the patients in the present study.

In this study, 12 patients were enrolled in the experimental group with comparable baseline characteristics as the control group (Table 1). The enrollment criteria were as follows: (1) over 18 years old; (2) within 4.5 h of disease onset; (3) paroxysmal or persistent motor hemiparesis without cortical symptoms and symptom duration greater than 1 h; (4) no contraindications to intravenous thrombolytics; (5) exclusion due to hemiplegic migraines and focal seizures; (6) magnetic resonance imaging (MRI) of the head within 24 h of onset; (7) no obvious alleviation (NIHSS score reduction < 4, tirofiban was administered immediately); (8) symptoms recurred within 24 h after thrombolysis (NIHSS increased by more than 4 points), and tirofiban was given for more than 1 h; (9) examination to exclude severe vascular stenosis or occlusion in responsible blood vessels by digital subtraction angiography (DSA) immediately after intravenous thrombolysis. Patients who meet one of the following conditions are excluded from this study: (1) definite cerebral hemorrhage determined by emergency head computed tomography (CT); (2) unstable vital signs; (3) complicated with severe functional impairment of other important organs; (4) those who do not meet the inclusion criteria of this study. Eight patients enrolled in the control group were the final diagnosed CWS who had previously received intravenous thrombolysis. The patients enrolled in the control group met the criteria listed as above (item 1–7). Those who had cerebral hemorrhage confirmed by emergency cranial CT were excluded in the present study.

**TABLE 1:** Comparison of clinical baseline data and modified Rankin Scales (MRS) score between recombinant tissue plasminogen activator (rt-PA) treatment and recombinant tissue plasminogen activator (rt-PA) combined with tirofiban groups, *n* (%).

Variable		rt-PA + tirofiban (*n* = 12)	Rt-PA (*n* = 8)	*P*-value
Sex	Male	9 (75.0)	6 (75.0)	1.000
	Female	3 (25.0)	2 (25.0)	
Age (x ± s)		62.2 ± 11.2	61.0 ± 10.2	0.815
Smoking	Y	8 (66.7)	5 (62.5)	1.000
	N	4 (33.3)	3 (37.5)	
Family history of stroke	Y	2 (16.7)	2 (25.0)	1.000
	N	10 (83.3)	6 (75.0)	
Previous ischemic stroke	Y	1 (8.3)	0 (0.0)	1.000
	N	11 (91.7)	8 (100.0)	
Hypertension	Y	11 (91.7)	6 (75.0)	0.537
	N	1 (8.3)	2 (25.0)	
Diabetes mellitus	Y	4 (33.3)	3.0 (37.5)	1.000
	N	8 (66.7)	5 (62.5)	
Hypercholesterolemia	Y	11 (91.7)	6.0 (75.0)	0.537
	N	1 (8.3)	2 (25.0)	
Transient ischemic attacks	Y	1 (8.3)	0 (0.0)	1.000
	N	11 (91.7)	8 (100.0)	
Atrial fibrillation	Y	1 (8.3)	1 (12.5)	1.000
	N	11 (91.7)	7 (87.5)	
Previous antiplatelet therapy	Y	0 (0.0)	0 (0.0)	1.000
	N	12 (100.0)	8 (100.0)	
Previous anticoagulant therapy	Y	0 (0.0)	0 (0.0)	1.000
	N	12 (100.0)	8 (100.0)	
Previous treatment with statins	Y	2 (16.7)	2 (25.0)	1.000
	N	10 (83.3)	6 (75.0)	
3-month MRS score M (P25, P75)		0.00 (0.00–0.00)	1.00 (0.25–1.75)	0.003

Y, yes; N, no.

## Treatment process

In accordance with medical ethics, some patients had the first onset of symptoms, but the symptoms lasted for more than 1 h, and the NIHSS score was higher than four. After cranial CT excluded cerebral hemorrhage, intravenous thrombolysis was administered. Based on clinical manifestations and imaging features, CWI was finally diagnosed. Tirofiban treatment regimen was as follows: patients were injected intravenously with tirofiban (0.4 μg/kg/min) for the first 30 min, followed by intravenous infusion (0.1 μg/kg/min) for 24–72 h depending on the timeframe of symptoms improvement. Overlapped with the last 4 h of tirofiban infusion, patients were given oral aspirin (100 mg) and clopidogrel (75 mg) each day continuously for 21–90 days (21 days for any case with mild stroke), and then oral aspirin (100 mg) or clopidogrel (75 mg) daily for continuous treatment afterward. Based on the results of routine CYP2C19 genetic test, the patients who had medium or slow metabolic rate were given a first dose of 180 mg of ticagrelor on day 1. After that, 90 mg twice a day starting on day 2 to replace clopidogrel for anti-thrombosis. In the control group, double antibodies were started 24 h after intravenous thrombolysis, and the specific double antibody scheme was the same as in the experimental group.

Other basic treatment included 10 mg of oral ruishufatadine per day after admission (substituted with 40 mg of oral atorvastatin instead for patients with intolerance or abnormal renal function) followed by 5 mg of oral ruishufatadine daily after 3 months. Patients’ blood pressure was stabilized at systolic pressure of 90–140 mmHg, with fasting blood glucose level at 4–7 mmol/L and postprandial level at 7–11 mmol/L. Patients were also given urinary kallidinogenase by intravenous drip for 7–14 days after admission.

### Statistical analysis

SPSS version 25.0 software (SPSS for Windows, IBM Corp., Armonk, NY, USA) was used for statistical analysis. The Kolmogorov–Smirnov test was used to assess the normal distribution of data. Age was described using the mean ± SD, student’s *t*-test was used to compare the age between the two groups; 3-month MRS score data was described using the median (interquartile range) M (P_25_, P_75_), the rank sum test was used to compare the 3-month MRS score between groups. Sex, smoking, family history of stroke, previous ischemic stroke, hypertension, diabetes mellitus, hypercholesterolemia, TIAs, atrial fibrillation, previous antiplatelet therapy, previous anticoagulant therapy, and previous treatment with statins data were expressed as *n*(%), and Fisher’s exact test was used for comparison between groups. Generalized estimating equation (GEE) model was used to identify the associations between different treatments and times with the NIHSS score. *P* < 0.05 was considered statistically significant.

## Results

### Comparison of clinical baseline and modified Rankin Scales score between recombinant tissue plasminogen activator treatment and recombinant tissue plasminogen activator combined with tirofiban groups

The study enrolled a total of 20 patients, including 12 in the experimental group and 8 in the control group. The study population consisted of nine males (75%) in the experimental group with an average age of 62.2 ± 11.2 years (range: 45–79 years), and six males (75%) in the control group with an average age of 61.0 ± 10.2 years (range: 44–74 years). Thus, there were no significant differences between the two groups in terms of sex or age (*P* > 0.05, Table 1).

The CWS-related risk factor and primary prevention did not differ significantly (*P* > 0.05) between the two groups. Electrocardiographic (ECG) monitoring was performed on all 20 patients for at least 48 h, and dynamic ECG monitoring was further given to those of abnormality, through which the case of paroxysmal atrial fibrillation in the experimental group [patient#9; 1/12 (8.3%)] and another case of persistent atrial fibrillation in the control group [patient#6; 1/8 (12.5%)] were detected. No significant difference was found between the two groups (*P* > 0.05, Table 1). Additionally, two atrial fibrillation patients did not start anticoagulant therapy during follow-up.

### Visible capsular infarction focus is detectable in the patients within 24 h of capsular warning syndrome onset while under intravenous thrombolysis

**FIGURE 1:**
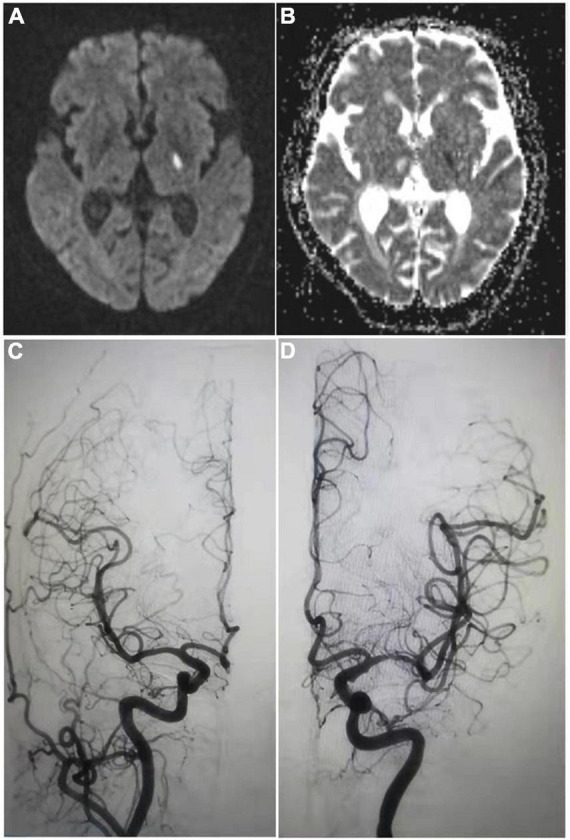
Visible capsular infarction focus is detectable in the patients within 24 h of capsular warning syndrome (CWS) onset while under intravenous thrombolysis. Shown are examples of imaging results from patient #5 of the experimental group. **(A)** The high signal of diffusion-weighted imaging (DWI) sequence **(B)** and low signal of apparent diffusion coefficient (ADC) sequence in internal capsule. The anterior circulation angiography under digital subtraction angiography (DSA) shows that panel **(C)** the lumen of the right middle cerebral artery (MCA) was well developed, and panel **(D)** the lumen of the M1 segment of the left MCA was slightly narrow with rough wall, indicating atherosclerotic changes.

Within 24 h of onset, all 20 study patients underwent MRI on the head with diffusion-weighted imaging (DWI) and apparent diffusion coefficient (ADC) calculations. Despite the absence of acute infarction focus in one case in the control group, all other patients had acute infarction focus within the internal capsule (Figure 1 and Supplementary Tables 1, 2). A vascular examination was performed on all 12 patients in the experimental group to identify responsible blood vessels during intravenous thrombolysis. Occlusion or severe stenosis in major blood vessels was excluded in all 12 patients. Among them, five cases (41.7%) were confirmed with various degrees of atherosclerosis accompanied by mild stenosis in their horizontal (M1) segment of middle cerebral artery (MCA; main blood vessel of lenticulostriate artery), and the remaining seven patients (58.33%) DSA results indicate lesions of the perforating branch of MCA (Table 2 and Figure 1). Among eight patients from the control group, five had routine magnetic resonance angiography (MRA) examination, three had head computed tomography angiography (CTA) scan (37.5%), and two were found with mild stenosis in M1 segment of MCA on the affected side (25%).

**TABLE 2:** Comparison of National Institutes of Health Stroke Scale (NIHSS) score between recombinant tissue plasminogen activator (rt-PA) treatment and recombinant tissue plasminogen activator (rt-PA) combined with tirofiban groups.

Variable	NIHSS score			*P*_1_ value
	
	Before thrombolysis	After thrombolysis	1 week after onset	
rt-PA + tirofiban (*n* = 12)	9.00 ± 1.71	6.00 ± 2.89	1.17 ± 1.70	<0.001
Rt-PA (*n* = 8)	8.88 ± 1.89	5.13 ± 2.10	5.13 ± 2.95	<0.001
*P*_2_ value	0.97	0.384	0.012	

P_1_ value means difference in time points within rt-PA combined with tirofiban group or Rt-PA treatment group; P_2_ value means rt-PA combined with tirofiban group vs. rt-PA treatment group at three separate times. NIHSS, National Institutes of Health Stroke Scale.

**FIGURE 2:**
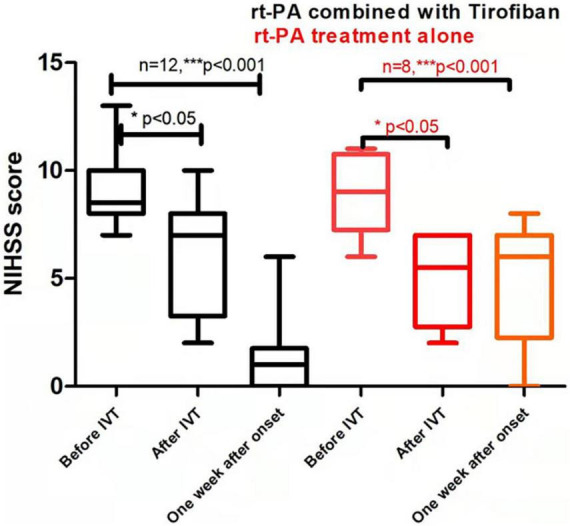
Comparison of clinical prognosis between recombinant tissue plasminogen activator (rt-PA) treatment alone/control group and rt-PA combined with tirofiban treatment/experimental groups. Values are reported as *M*(P_25_–P_75_). P value in the last column indicates the probability of obtaining test results after comparing rt-PA combined with tirofiban group vs. rt-PA treatment group alone. Asterisk indicates significant difference by comparing the scores after IVT or 1 week after onset with the scores before IVT of the same group, *P < 0.05; **P < 0.01; ***P < 0.001. Parenthesized asterisk indicates significant difference by comparing the scores 1 week after onset with the scores after IVT of the same group, *P < 0.05; **P < 0.01; ***P < 0.001.

In terms of treatment efficacy, 20 patients had at least four recurrent attacks. Overall, symptoms were stable for all 12 patients in the experimental group from the start until the end of tirofiban use, without any case of worsening symptom after tirofiban treatment had stopped (0%). The 12 patients in the experimental group had a favorable clinical outcome with median MRS scores of 0 by 3-month. Among the eight control patients, four experienced fluctuations of symptoms since hospitalization (50%), while another four had obvious improvements within a week after intravenous thrombolysis (50%) (Figure 2 and Supplementary Tables 1, 2). By 3-month, 87.5% (7/8) of control group patients had a medium score of 1 on the MRS of favorable prognosis. In both experimental and control groups, the significant reduction of NIHSS scores were found after intravenous thrombolysis or 1-week after onset compared with the score before intravenous thrombolysis (*P* < 0.01). In addition, there were no differences between the experimental and control groups before or after intravenous thrombolysis (*P* = 0.970, *P* = 0.384, respectively). In contrast, both 1-week NIHSS and 3-month MRS scores in the experimental group were significantly lower than those in the control group (*P* = 0.012, *P* = 0.003, respectively; Figure 2, Table 2, and Supplementary Tables 1, 2). Only one patient in the experimental group (patient #8) did not show a substantial relief of symptoms after intravenous thrombolysis followed by tirofiban treatment. By contrast, the rest of the patients all had various degrees of improvement in their symptoms. Overall, a significant reduction in NIHSS scores was observed in the experimental group after the administration of tirofiban when compared with the scores recorded before the administration of tirofiban (Figure 2).

In terms of safety, there were no symptomatic intracranial hemorrhages in any of the patients during treatment. Tirofiban dosage and treatment time were determined based on the alleviation and fluctuation of symptoms in the experimental group of patients. The average dose of tirofiban was 17.00 mg with a maximal dose of 32.63 mg (patient #11) for a maximum of 72 h. No obvious systemic hemorrhage was detected in those patients, despite one case (patient #5) presenting with hemorrhage in the digestive tract. Nonetheless, the symptom was alleviated in that patient after the suitable treatment without ceasing tirofiban use. There was one case (patient #2) in the control group with hematuria during intravenous thrombolysis, which may present as a result of trauma in the urothelial mucous membrane during catheter insertion and was resolved after proper treatment without causing fatal hemorrhage (Supplementary Tables 1, 2). Additionally, none of the patients in the study experienced peripheral thrombocytopenia throughout the treatment (meaning platelet count reduced to <100 × 10^9^/L).

## Discussion

Capsular warning syndrome has a low disease incidence with a high tendency to develop into cerebral infarction. Most cases in our study were diagnosed with acute capsular infarction and verified by MRI with a TIA incidence of 5% (1/20) at the end. The average age of disease onset in the study subjects was 61.6 ± 6.9 years, and 75% of them were male, which resembles demographics reported in the literature regarding age of onset and gender ratio (Donnan et al., 1993; Camps-Renom et al., 2015). There have been a few hypotheses on the physiological mechanisms for pathogenesis of CWS, and the current consensus of most studies is that the key mechanisms are related to BAD lesion, arteriola hyalinization caused by atherosclerosis in the main artery, changes, and exhaustion in hemodynamics (Lalive et al., 2003). Hence various clinical treatment strategies are adopted, such as intravenous thrombolysis, anticoagulation, dual antiplatelet oral medication, and increase of blood pressure/perfusion. However, despite different treatment approaches, CWS has an unsatisfactory clinical prognosis (Paul et al., 2012).

Recombinant tissue plasminogen activator (also known as alteplase) is a highly selective, effective, and second-generation intravenous thrombolytic drug with the properties of fibrinogen, which makes it the first-choice medicine for intravenous thrombolysis (Tung et al., 2017). rt-PA, however, can easily activate platelet aggregation, promote the breaking of thrombi, and clog blood vessels (Tassi et al., 2013; Tsivgoulis et al., 2017). In addition, it does not alter the pathological condition of the vascular wall, nor does it restore the smooth surface of the vascular endothelium or reverse hypercoagulability. The results of the control group (intravenous thrombolysis with rt-PA alone) show that half of the patients still experience recurring symptoms or progress. As a result, neither traditional dual antiplatelet drugs nor rt-PA-based intravenous thrombolysis have proven effective in treating CWS (Gutiérrez Ruano et al., 2013).

In the Berge et al. (2021) published a new guideline on intravenous thrombolysis for AIS with the following recommendation: “For patients with acute ischemic stroke of <4.5-h duration, we recommend no antithrombotic drugs within 24 h of intravenous thrombolysis over antithrombotic drugs as an adjunct therapy to intravenous thrombolysis with alteplase (Berge et al., 2021)”. However, platelet GPIIb/IIIa receptor is the final common pathway of platelet aggregation and thrombosis. Tirofiban is the most widely used GPIIb/IIIa receptor antagonist in China. GPIIb/IIIa receptor is expressed on the surface of platelets and megakaryocytes. As the final pathway of platelet aggregation, it connects adjacent platelets by binding to fibrinogen. Tirofiban is dose dependent, and its binding mode with GPIIb/IIIa receptor is reversible. It can be used flexibly and has high safety (Coller, 2001; Schwarz et al., 2006; Dornbos et al., 2018).

According to literature reports, CWS is considered as a special type of TIA. Epidemiological studies have found that CWS only accounts for 1.5–1.8% of all TIAs (Yu et al., 2020). Its clinical characteristics and diagnostic criteria generally include basic diseases with atherosclerosis, accompanied by dyskinesia of movement, sensation and articulation, most symptoms are repeated and progressive, imaging shows that there is or is no disease in the internal capsule area, and no obvious abnormality is found in the intracranial large vessels (Zhu et al., 2021).

Wu et al. also reported that low dose tirofiban improved the prognosis of neurological function after intravenous thrombolysis (Wu et al., 2019). They included AIS patients whose symptoms deteriorated after intravenous thrombolysis, with pathogenesis including atherosclerosis in the main artery, occlusion in minute vessels, cardiogenic embolism, and other unknown factors. Patients in that study were given an average dose of tirofiban at 6.5 ± 1.9 mg and not exceeding 24 h of intravenous thrombolysis. Their results indicate a favorable clinical prognosis rate of 57%, which is significantly higher than that of the control group. At the end of study, they concluded that it would be safe and effective to administer low dose tirofiban early on in AIS patients within 24 h of intravenous thrombolysis. Therefore, our study adopted tirofiban as antiplatelet therapy following intravenous thrombolysis to improve the clinical efficacy of treating CWS.

The pathogenesis of the patients enrolled in this study was mostly due to the occlusion or alteration of hemodynamics resulting from pathological changes of perforator artery, rather than cardiogenic embolism or pathological changes with severe stenosis in the main artery. Based on clinical observation, most of the patients were with fluctuating symptoms stabilized within 2–3 days of tirofiban treatment. In addition, there were no incidences of hemorrhage directly related to tirofiban administration. Therefore, prolonged use of tirofiban for treating CWS triggered by atherosclerosis in branch arteries or arteriola hyalinization should be quite safe.

Early administration (within 24 h) of antiplatelet treatment has been a perplexing clinical problem. The present study is the only study using tirofiban immediately following 24-h intravenous thrombolysis. However, there are limitation to this study. First, the data may be biased due to the small number of patients enrolled in the experiment group. Secondly, fluctuation of clinical symptoms (NIHSS scores) and tirofiban infusion time were determined at the discretion of the physician in charge hence were they determined by individual judgment. The findings do suggest that, in this small open, no randomized sample with historic controls, tirofiban given after rt-PA may be safe and possible efficacious. The warrants further study in the form of a large randomized controlled trial.

## Conclusion

This study is unique in that we continuously treat CWS patients who failed to benefit from rt-PA intravenous thrombolysis with tirofiban. In addition, the use of tirofiban early after intravenous thrombolysis can achieve better efficacy and improve patients’ neurological function, as well as being safe. Due to the small number of samples included in this study, the sampling may be insufficient due to obvious regional characteristics. Taken together, our results demonstrate that tirofiban has great clinical value for continuous subsequent treatment in CWS patients with poor prognosis for intravenous thrombolysis.

## Data availability statement

The raw data supporting the conclusions of this article will be made available by the authors, without undue reservation.

## Ethics statement

The studies involving human participants were reviewed and approved by the Third People’s Hospital Affiliated to Fujian University of Traditional Chinese Medicine. Legal representative is Jianhong Chen. The patients/participants provided their written informed consent to participate in this study.

## Author contributions

MZ: study conception and design. YL, SL, and YY: acquisition of data. ZZ: interpretation and collection of image data. DH, WL, SL, and YL: analysis and interpretation of data. All authors drafting the manuscript and final approval of the version to be published.
