# Supplementary material for: Clinical safety and possible efficacy of tirofiban in combination with intravenous thrombolysis by recombinant tissue plasminogen activator for early treatment of capsular warning syndrome (CWS)
Source: Front Neurosci. 2022 Nov 16;16:1026127. doi: 10.3389/fnins.2022.1026127 (PMC9709144; doi:10.3389/fnins.2022.1026127)
Supplement: Supplementary file 1 [file Table_1.DOCX]

**Table S1.** Clinical data of rt-PA combined with Tirofiban treatment/experimental group

| Patient | Sex/  age (year) | | Risk factors | DSA  results | Times of symptom recurrence | NIHSS score before IVT | NIHSS score after IVT | IL  found on MRI | Degree of symptom change（NIHSS） and within 24 hours from the end of thrombolysis（score/mins） | NIHSS score after Tirofiban use | Tirofiban dose  (mg)  /time  (hour) | NIHSS score one week after onset | MRS score 3 months after onset | Severe bleeding complications |
| --- | --- | --- | --- | --- | --- | --- | --- | --- | --- | --- | --- | --- | --- | --- |
| 1 | M/ 57 | S, Ht, Hc | | AS-RMCA M1(＜30%) | 3 | 10 | 4 | PLIC | 9/120 | 3 | 9.95/  24 | 1 | 0 | N |
| 2 | M/ 45 | S, FHS, Ht, Hc | | PVO | 1 | 8 | 8 | PLIC | 8 | 3 | 10.71/  24 | 1 | 0 | N |
| 3 | M/ 75 | P, Ht, Hc, TIA | | PVO | 1 | 10 | 10 | PLIC | 10 | 5 | 20.58/  48 | 0 | 0 | N |
| 4 | M/ 65 | S, D, Hc | | AS-LMCA M1(＜30%) | 1 | 9 | 8 | PLIC | 8 | 5 | 22.64/  48 | 2 | 0 | N |
| 5 | F/ 54 | S, Ht, Hc | | AS-LMCA M1(＜30%) | 1 | 7 | 7 | PLIC | 7 | 0 | 8.43/  24 | 0 | 0 | N |
| 6 | M/ 79 | Ht, D, Pts | | PVO | 2 | 8 | 4 | PLIC | 8/610 | 5 | 11.93/  24 | 2 | 0 | N |
| 7 | M/ 66 | S, FHS,Ht, Hc | | AS-RMCA M1(＜30%) | 2 | 10 | 2 | PLIC | 8/320 | 4 | 29.58/  72 | 0 | 0 | N |
| 8 | M/ 50 | S, Ht, Hc | | PVO | 2 | 13 | 7 | PLIC | 11/45 | 8 | 20.69/  48 | 6 | 1 | N |
| 9 | F/ 70 | S, Ht, Hc, AF, | | PVO | 2 | 7 | 2 | PLIC | 8/95 | 4 | 8.62/  24 | 1 | 0 | N |
| 10 | M/ 72 | Ht, D, Hc, Pts | | AS-RMCA M1(＜30%) | 1 | 10 | 10 | PLIC | 10 | 4 | 11.17/  24 | 1 | 0 | N |
| 11 | M/ 48 | S, Ht, Hc | | PVO | 1 | 8 | 7 | PLIC | 7 | 0 | 32.63/  72 | 0 | 0 | N |
| 12 | F/ 65 | Ht, D, Hc | | PVO | 2 | 8 | 3 | PLIC | 7/700 | 3 | 17.05/  48 | 0 | 0 | N |

Abbreviations: M, male; F, female; S, smoking; FHS, family history of stroke; P, previous ischemic stroke; Ht, hypertension; D, diabetes mellitus; Hc, hypercholesterolemia; TIA, transient ischemic attacks; AF, atrial fibrillation; Pap, previous antiplatelet therapy; Pac, previous anticoagulant therapy; Pts, previous treatment with statins; DSA, digital subtraction angiography; AS, atherosclerotic stenosis; PVO, perforating vessel occlusion; RMCA, right middle cerebral artery; LMCA, left middle cerebral artery; N, negative; Y, yes; IVT, intravenous thrombolysis; IL, infarct location. PLIC: Posterior limb of internal capsule.

**Table S2.** Clinical data of rt-PA treatment alone/control group

| Patients | Sex/age (y) | Risk factors | Times of symptom recurren-ce | NIHSS score before IVT | NIHSS score after IVT | IL found  on MRI | Degree of symptom change（NIHSS） and within 24 hours from the end of thrombolysis（score/mins） | NIHSS score one week after onset | MRS score 3 months after onset | Severe bleeding complications |
| --- | --- | --- | --- | --- | --- | --- | --- | --- | --- | --- |
| 1 | M/  71 | S, Ht, D, Hc | 3 | 7 | 7 | PLIC | 7 | 8 | 3 | N |
| 2 | M/  54 | S, Ht, Hc | 2 | 8 | 5 | PLIC | 8/110 | 6 | 1 | N |
| 3 | F/  44 | P, Hc | 4 | 11 | 5 | PLIC | 10/400 | 7 | 2 | N |
| 4 | M/  61 | S, D, Hc | 1 | 8 | 6 | PLIC | 6 | 6 | 1 | N |
| 5 | F/  74 | S, FHS, Ht, Hc | 2 | 10 | 7 | PLIC | 10/300 | 7 | 1 | Urethral hemorrhage |
| 6 | M/  70 | Ht, D, Pts,  AF | 1 | 6 | 2 | N | 2 | 1 | 0 | N |
| 7 | M/  56 | FHS, Ht, Hc, Htc | 1 | 10 | 2 | PLIC | 2 | 0 | 0 | N |
| 8 | M/  58 | S, Ht | 1 | 11 | 7 | PLIC | 7 | 6 | 1 | N |

Abbreviations: M, male; F, female; S, smoking; FHS, family history of stroke; P, previous ischemic stroke; Ht, hypertension; D, diabetes mellitus; Hc, hypercholesterolemia; TIA, transient ischemic attacks; AF, atrial fibrillation; Pap, previous antiplatelet therapy; Pac, previous anticoagulant therapy; Pts, previous treatment with statins; IVT, intravenous thrombolysis; IL, infarct location; Y, yes; N, negative. PLIC: Posterior limb of internal capsule.
